# Immigration, Statecraft and Public Health: The 1920 Aliens Order, Medical Examinations and the Limitations of the State in England

**DOI:** 10.1093/shm/hkv139

**Published:** 2016-04-15

**Authors:** Becky Taylor

**Keywords:** public health, state, aliens, immigration, governance

## Abstract

This article considers the medical measures of the 1920 Aliens Order barring aliens from Britain. Building on existing local and port public health inspection, the requirement for aliens to be medically inspected before landing significantly expanded the duties of these state agencies and necessitated the creation of a new level of physical infrastructure and administrative machinery. This article closely examines the workings and limitations of alien medical inspection in two of England’s major ports—Liverpool and London—and sheds light on the everyday working of the Act. In doing so it reflects on the ambitions, actions and limitations of the state and so extends research by historians of the nineteenth and early twentieth century on the disputed histories of public health and the complexities of statecraft. Overall it suggests the importance of developing nuanced understandings of the gaps and failures arising from the translation of legislation into practice.

## Introduction

The 1919 Aliens Act and subsequent 1920 Aliens Order are little-studied pieces of legislation, but for those who have made them the subject of their research they are notorious.^1^ Described in damning terms by contemporaries and historians alike, the Act formed the bedrock of Britain’s inter-war immigration policy, and was characterised by the Home Secretary of the time, Edward Shortt, as both ‘stringent and … wide’.^2^ At its core was the presumption that the state should, ‘exercise complete and absolute control over the admission of aliens … either individually or in classes, to supervise them during their stay here, and to require them to leave the country if and when … desirable’.^3^ David Cesarani attributes to it the ‘systematic expulsion’ of over 30,000 ‘enemy aliens’ and highlights the ways in which the legislation was used to express anti-Semitism and to expel foreign trade union radicals and labour activists. Others have observed that it removed the category of refugee from immigration law—which had been included in the Aliens Act 1905—and have pointed to the role of ministerial discretion in the appeals process in constructing an ‘illiberal and arbitrary regime’.^4^ In addition, although the legislation excluded seamen from its terms, Laura Tabili’s work has emphasised how it laid the groundwork for the Coloured Alien Seamen Order of 1925.^5^

Overlooked in discussions of the measure to date has been the place of medical inspection in the Aliens Order. Building on the terms of the Aliens Act 1905, the Order required that all aliens entering Britain be medically inspected and allowed immigration officers to refuse entry to those suffering from particular conditions.^6^ While there was an established tradition of controlling the entry of disease via Britain’s ports—from 1825 through quarantine and after 1872 through the ‘English system’—such measures were based on medical criteria and were concerned with treating and supervising infected persons and not with immigration control.^7^ The Aliens Acts instead presumed that a certain class of immigrant—aliens—presented identifiable and specific health risks to the British population and therefore should be controlled.^8^ Consequently the 1920 Aliens Order can be understood as being more influenced by contemporaneous developments in the United States and Australia, which were concerned with patrolling the entry of immigrant groups seen as inherently more diseased and undesirable, than by British nineteenth-century public health practices.^9^

While the aim of controlling the movement of international migrants was a product of the unprecedented explosion in European transatlantic migration from the mid-nineteenth century, it was equally the result of shifting ideas of what states were *for* and what they might be able to *do*. Victorian statecraft has been a lively area of historical enquiry, with studies ranging from the failures of initial smallpox vaccination campaigns to the endemic malfunctioning of the Local Government Board demonstrating the partial and contested nature of late nineteenth-century state expansion.^10^ The twin themes of the successes and limitations of governmentality, and histories of the expansion of public health have also been extended into considerations of the first half of the twentieth century.^11^

This article extends the existing historiography in three particular directions. First, through focusing on Liverpool and London, it explores how medical immigration policy was translated into practice at Britain’s two busiest migrant ports. Secondly, through using correspondence between the ports, the Home Office and the newly created Ministry of Health alongside detailed Ministry inspection reports, it places this emerging alien medical inspection regime within the expansion of a modern bureaucratic state. More work remains to be done on the local political dynamics of the Order in these and other major ports, but this article keeps as its focus the relationship between central and local government. In doing so it draws together Foucauldian understandings of the diffuse nature of state power and the work of Levene *et al.*, Taylor *et al.* and Welshman. Together these historians have pointed to the importance of understanding the interplay of power dynamics between Whitehall and the localities; the respective and competing influence of different government departments; and the role of particular officials in enacting public health measures.^12^ Building on these concerns, I argue that the prism of public health can be used to explore larger questions around statecraft and governmentality in the first decades of the twentieth century while also providing an insight into the everyday workings of an emerging state structure.

Thirdly, and crucially, the narrowing of entry rights to Britain over the course of the twentieth century has meant that histories of the regulation of immigration into Britain have been commonly constructed as a story of the inexorable extension of state control and tighter restrictions.^13^ As a result, while some historians have pointed to the largely ineffective nature of the 1905 Act, overall histories have tended to be written with the presumption that subsequent immigration legislation was unproblematically enforced.^14^ Challenging this, David Feldman has argued that the British government’s record throughout the twentieth century was notable more by its failure to control immigration to its shores than it was by successful restrictionism.^15^ This article also contributes to this debate by demonstrating how the 1920 Aliens Order might contribute to a broader history of ineffective immigration control.

## Immigration, Statecraft and Public Health

The first years of the twentieth century saw mass emigrations from eastern and central Europe to France, Britain and on to the Americas. This coincided with and drove nation states’ increasing preoccupation with the need to codify who belonged and counted as a citizen.^16^ Concurrent with the consequent growing state control of alien immigration was an explosion of interest—state, private and professional—in public health issues and in understanding the interrelationship between personal, local, national and international factors in causing and preventing infectious disease. There were long-established international practices allowing for the quarantine of infected and suspected ships, goods and persons, and the late nineteenth century saw intense international debate over disease control and the patchy emergence of international cooperation.^17^ Alongside national and bi-partisan arrangements, the international Sanitary Conferences and the creation of the *Office international d’hygiène publique*, although producing few tangible results, had allowed a network of experts to emerge and actively discuss internationally recognised standards of infectious disease control.^18^ Recent research has increased our understanding of how the hardening of national, imperial and international administrative boundaries was often intimately bound up with concerns over policing public and ‘racial’ health to construct racialised *cordon sanitaires*.^19^ The growth of immigration control to the United States and its requirement for immigrants to be certified as free from disease from 1882 was the most prominent international example in the northern hemisphere, and one which was to have major repercussions across the Atlantic in how emigrants were processed in ports of departure.^20^

However, Maglen’s work on the ‘English system’ demonstrates the fallacy of simplistically transposing Australian and American experiences to other nations. In arguing that the nineteenth-century British government’s preoccupation with safeguarding and promoting trade within and beyond the empire produced an elastic notion of British borders, she shows that factors other than ‘race’ or nationhood shaped emerging systems of immigration control. She also demonstrates how, in contrast to the types of public health arguments made against immigrants in key nations of immigration, Britain’s anti-alien discourses were shaped primarily around sweated labour, overcrowding of working class neighbourhoods and generalised fears of being ‘swamped’ by immigrants.^21^ Indeed, despite the role of migrants in the 1892 cholera outbreak, Port Sanitary Authorities emphasised the robustness of their existing system of isolation and surveillance, refusing to single out aliens as a particular threat. Evidence Medical Officers gave to the Royal Commission on Alien Immigration in 1902–03 stressed that risks posed by immigrants to the nation’s health was minimal provided that there was sufficient information about their identity and intended destination. That the resulting Aliens Act 1905 contained specific medical provisions was the product of the vociferous anti-alien campaign and the growing impact of increasingly stringent American immigration laws, and not, Maglen shows, a product of public health lobbying. This was reflected in the Act’s wording, which singled-out conditions which might cause an immigrant to become a charge on the rates rather than on perceived threats to public health.^22^ Overall, Maglen’s analysis of the 1905 Aliens Act usefully situates the expanded role of Port Medical Officers within the emerging tradition of the English System and its preoccupation with minimising inconvenience to those involved in trade and commerce. While her work reinforces the well-established point that much public health and immigration legislation was strongly bound up with classed notions of disease, and points to the limited impact of the legislation—noting that only eighteen immigrants were deported on medical grounds in the first year of the Act—her analysis does not extend forward to consider the 1920 Aliens Order.^23^

In part the 1919 legislation can be seen as emerging from key developments of the nineteenth century. Yet it also needs to be placed within its immediate historical context. The early twentieth century anti-alien legislation had had the effect of ‘breaking the Victorian inhibition on the subject of restriction of immigration and familiarising the public mind with the idea that a country had the right to keep out unwanted immigrants if it chose’.^24^ By the end of the First World War both the control of aliens and the role of public health surveillance in managing the flow of people through its ports had become accepted parts of state activity. Consequently, the 1919 Act—prompted by a combination of administrative pressure and a calculated attempt by the Coalition to capitalise on popular anti-alien sentiment—was underpinned by an acceptance that not only did a state have the right to exclude certain people, but it had the means for doing so.^25^ However this assumption rubbed up against a number of constraints: financial, practical and political.

Building on scholarship for the nineteenth century, historical work has shown the difficulties experienced by inter-war attempts to translate policy into practice. While Britain in the 1920s had a relatively sophisticated bureaucracy, and although there was broad acceptance that the government’s role extended beyond the minimalism of the early nineteenth century laissez-faire state, its reach and functions remained shifting and contested territory.^26^ The expansion of the state after 1918, including the creation of the Ministry of Health, formed part of a broader process of post-war reconstruction encompassing health, housing, education and employment. The Coalition’s measures were wide-ranging and were as much concerned with forestalling industrial and political unrest as with genuine reform, so that both the creation of the Ministry and the passing of anti-alien legislation can be understood in this context.^27^

The Ministry of Health’s first Permanent Secretary, Robert Morant, saw its creation as an opportunity to ‘bring in new blood, give a “lift” to the whole corps, develop dynamic energy throughout it, and infect the secretariat too, and generally give enhanced prestige to the whole Ministry.’^28^ But as Stacey argues, Morant’s unexpected death in 1920, coupled with Addison’s political demise and the Geddes Axe of 1922, which required all departments to engage in significant retrenchment, simultaneously reinforced the dominance of the Treasury and truncated plans for a dynamic and forward looking Ministry of Health.^29^ Taylor and colleagues have concluded that despite some local authorities’ enthusiasm for taking full advantage of powers granted them under the permissive public and personal health acts passed in 1916–19, their ambitions were severely restricted by retrenchment and were actively constrained by Ministry officials.^30^ And so, while after 1918 there were significant extensions in the functions of the state we cannot assume either that this saw the simultaneous creation of the necessary administrative machinery, nor that central and local government acted in concert to implement the new measures.^31^ This insight has yet to be mapped onto the workings of Britain’s immigration control practices, but suggests that both the Home Office and the Ministry of Health might be fruitfully explored in this context.^32^

In the first years of the twentieth century ports were the first place where incoming travellers met the different faces—customs officials, immigration officers and medical inspectors of aliens—of this expanding state. Foucault first called attention to the importance of unpicking both the genealogy of statecraft and its bio-politics in order to reveal the means by which states physically control their own populations and exclude other populations.^33^ This, he argued, opens up understandings of the diffused nature of power, how it is embodied and enacted within societies and how it operates in relation to the organisation and control of space. He placed particular emphasis on the ‘heterogeneous and dispersed microphysics of power’, and the importance of examining ‘specific forms of its exercise in different institutional sites, and … where it is exercised over individuals rather than legitimated at the centre’.^34^ Diffusion and dispersion, however, might also translate into dilution, particularly in the context of inadequate funding and resources. So, this would suggest while borders are places where state power is imposed and performed, it is equally possible that borders might see its absence or weakness, complicating or destabilising face-to-face encounters between incomers and agents of the state.^35^

The key state figure in these face-to-face encounters at the border was the Port Medical Officer of Health (MOH).^36^ Although central to the expansion of personal and public health care in the inter-war period, MOHs remained the poor cousin within the medical establishment, looked down upon by the wider medical profession and often undervalued by the councils employing them.^37^ And within this already marginalised sector of the profession, the Ministry of Health recognised the particularly low status of those working within Port Sanitary Authorities (PSAs):

[F]ew people realise that such a service exists and still fewer have any grasp of its importance. … As there is such widespread ignorance and apathy on the part of the PSAs it is essential that their officers should be efficient, particularly as the PS Administration is the country’s first line of defence against such diseases as typhus, plague, small pox and against unwholesome foodstuffs.^38^

This reveals the contradiction between the potential importance of the role of Port MOHs on the one hand and their marginal status on the other. Part of the way in which medical officers’ status was signalled was via the material resources and infrastructure allocated to their work. The interwar expansion of the local state found physical expression in the construction of all sorts of municipal buildings—council housing, infant and child welfare clinics, tuberculosis and venereal disease clinics, general and isolation hospitals—which could be architecturally innovative and whose form reflected progressive councils’ aspirational politics.^39^ In common with other expanding areas of local authority responsibility, implementing the Aliens Order meant constructing new facilities—inspection rooms, medical offices and testing facilities—alongside employing new or specialised staff. In light of the potentially undervalued status of Port MOsH, this article consequently explores how the construction of facilities fitted into the newly expanding state.

Before the Aliens Order was put into effect the Ministry of Health needed to define exactly which diseases might lead to exclusion; then, via the relevant local authorities it needed to ensure there were medical officers on hand, and the necessary infrastructure in place, to examine incoming aliens. While the first of these was decided relatively quickly, the second required far more effort on the part of the state. In the remainder of this article I begin by showing how the Ministry of Health drew up the terms under which port medical inspectors acted. Then, through using correspondence with and reports generated by the Ministry of Health inspectors I move on to demonstrate how material, local, political and contingent factors mediated the workings of the Order in Liverpool and London.

## Defining the Unsanitary Alien

Legislation needs to be translated into working policy and this process necessarily involves interpretation. For the Aliens Order this was done by a Ministry of Health sub-committee of senior civil servants with one Home Office representative.^40^ Superficially, the Order was clear, aiming simply to secure the exclusion of aliens:

1. whose presence is likely to be a danger to the health of the people of this country, or;
2. who are likely to become a charge upon public funds, either by reason of their existing or probable future incapacity to support themselves and their dependents or because their condition is such as to render it probable that they will need treatment and care which they are unable to provide [from] their own resources.^41^

While the first of these could be assessed on purely medical grounds, the criteria governing the second, while obviously building on the terms of the 1905 Act, was far less clear-cut and seen by the Committee as ‘politically controversial’. In order to side-step controversy, officials argued that Medical Inspectors ‘should be restricted specifically to the ascertainment of *strictly* medical facts, and the offering of a *strictly* medical opinion on such medical facts’. Such concerns resulted in the Committee devoting considerable attention to defining the medical grounds for excluding an alien. Although generating technical advice for port medical officers was necessary, officials also argued that developing clear guidelines would ‘secure uniformity of action and *avoid questions* arising in individual cases’.^42^ Having formed their medical opinion, Medical Inspectors’ only other duty was to inform the Immigration Officer, ‘*on whom should fall the whole of the responsibility* for determining as to the landing or not landing of the alien’.^43^ Here we see an insistence on medical officers’ neutrality which fed into the Ministry’s broader claims of professional impartiality. In tandem with avoiding unfavourable attention, this was key for a new government department trying to establish its reputation. As we shall see, this position had practical ramifications, in that it suggested a close working relationship—physical and professional—between Immigration Officers and Medical Inspectors.^44^

The focus on medical criteria served to create an image of an impartial and professional alien inspection service. However, in the advice generated by the sub-committee it is already possible to see slippage between the ideal of alien control envisaged by the Home Secretary and practice on the ground. Article 16 (2) of the Order was clear in that the appointed medical inspector at a port ‘*may* inspect any alien seeking to land in the United Kingdom’, while Home Office advice made it clear it did ‘not consider it practicable or necessary that *every* alien coming to this country should be examined’. Only those coming for long periods, or temporary visitors where the Medical Inspector considered that ‘special circumstances require it’, needed to be examined.^45^ The introduction of ‘may’ into the wording, as well as the admission that it was practically impossible to inspect all aliens, legitimised a more limited approach to medical inspections and introduced an element of personal discretion. The 1905 Act’s much criticised requirement for only steerage passengers to be inspected might have been removed, but in practice ‘discretion’ would ensure that established class-based biases would continue to operate. This made it entirely feasible for first or second class aliens with a venereal disease, for example, to escape notice, while a steerage passenger with the same condition would be more likely to be refused entry.

Moreover, as the sub-committee developed its guidance, civil servants grappled with the range of physical and practical problems which a medical officer might face in carrying out their duties:

wherever practicable, the examination should take place on shore and during the hours of daylight … the Medical Inspector should have available the services of a trained nurse who should be present at all examinations of women on shore, and wherever practicable, on board ship also.

Here, while setting out the ideal, the use of conditional and aspirational language signalled civil servants paving the way for a more pragmatic approach. From the outset, the Ministry accepted how medical inspections might often need to take place on board ship even if it was ‘obvious that in some cases a medical examination under such circumstances must be unsatisfactory’.^46^

Overall then, while the 1920 Order was part of the first wave of measures for which the new Ministry of Health was responsible, from the beginning civil servants sought to introduce a restricted, even timid, working of the legislation. They were careful to emphasise the purely medical nature of the work and to distance themselves from political controversy. The sub-committee acknowledged both the partial nature of any potential inspection regime—it excluded seamen, imperial subjects and transmigrants—as well as introducing a discretionary element on the part of the medical inspector.

If the Ministry of Health rapidly accepted a highly constrained version of the Order’s original intention, how did this play with the Home Office, which was keen to build on war-time measures to ensure ongoing control of aliens?^47^ While demanding more restrictions, mindful of the contentious nature of alien immigration control, Haldane Porter, head of the Aliens Department was anxious to keep powers as low key as possible. It is this tension which explains both the wide ranging nature of the Order, and the fact that, like some other pieces of controversial legislation, it was renewed on an annual basis rather than being placed permanently in statute.^48^ Consistent with its stated ethos of professionalism, the Home Office argued publicly that annual renewal was necessary to allow it the necessary experience of regulating immigration in peacetime before any permanent legislation was passed.^49^ However, the internal Aliens Department history put a very different slant on the department’s actions, arguing that the process of asking for permanent legislation would risk exposure in the House of Commons debating chamber with the possible result of loss of all powers of alien control:

If it came up for discussion it is quite possible that the House, with its traditional dislike of vesting unfettered power in Ministers, would refuse to sanction it as a permanent measure. *Quieta non Movere* has therefore been the policy of the Home Office.^50^

Not then a case of impartial professionalism; rather a desire to continue authoritarian measures on a rolling temporary basis than risk losing them by going to Parliament for permanent legislation. Added to this was the fact that despite its ambitions, internal evidence demonstrates the Alien Department’s systemic inability to keep track of aliens. Notwithstanding Cesarani’s claims of the number of deportations carried out under the Aliens Order, evidence from the Home Office’s own files unequivocally demonstrates that it was incapable of maintaining either a comprehensive or up-to-date Aliens Register.^51^ While not directly impinging on the implementation of alien medical inspection, but in direct contrast to the American experience, this bureaucratic failure served as a background context in which the state was neither as efficient nor wide-ranging as its legislative ambitions suggested.^52^

From the perspective of central government, keeping the legislation on an annual rolling basis reduced its visibility, thus potentially allowing the Home Office to maintain its power. Yet, these same features actively inhibited civil servants from being able to claim more resources to invest in the proper implementation of the Order. In turn the Aliens Department’s unwillingness to develop permanent legislation combined with economic stringencies of the period and the narrowness of the Ministry of Health’s definitions to profoundly shape the implementation of the 1920 Order. This is revealing of the tension between different forms of power—the Order remained in force, but without the necessary financial commitment to make it fully workable. Thus power remained located in Whitehall—specifically within the Home Office—and not diffused to the localities. How this was to affect medical inspection regimes in two of England’s busiest passenger ports, Liverpool and London, is the subject of the remainder of the article.

## Liverpool

By the interwar period much of the transatlantic transmigrant traffic had shifted to Southampton, but nevertheless Liverpool remained a key part of the movement of emigrants from mainland Europe. It also, of course, continued to be central to flows of Irish migrants—described by officials as not ‘generally of a very good class’—those coming to England and Scotland as well as those going on to, or returning from America.^53^

While not initially taking up its powers as a Port Sanitary Authority under the 1872 Act, a severe cholera outbreak in 1892 had resulted in Liverpool appointing a Port MOH. From that point it developed a rigorous inspection regime under its energetic MOH, Edward Hope. Hope had significant professional and personal standing in the city as well as being active within national and international public health circles.^54^ Evidence from elsewhere has suggested that an MOH might be able to translate high professional standing into increased resources for their public health department.^55^ In contrast, Liverpool’s enforcement of the 1920 Order reveals the circumscribed nature of the MOH’s influence, and instead the centrality of material and physical constraints in governing medical inspectors’ ability to carry out their duties. Equally, Liverpool confirms the importance of setting the development of alien inspection within local, national and international scales of operation.

Publicly, the picture painted by Hope suggested one of modern efficiency and ‘minimum delay’, in which the co-operation between the different authorities—the Mersey Docks and Harbour Board, the Port Sanitary Authority and the Mersey Pilots—and the use of modern technology ensured a seamless response: ‘The pilots send wireless messages to the Port Sanitary Authority, which are passed on to the Medical Officer on duty, day or night, who boards the vessels immediately’.^56^

This may have been the case for cargo vessels and the established processes of the English System, but records of on-board medical inspection on passenger ships give a very different impression. In order to speed up inspection, immigration officers boarded at Queenstown in Ireland, and conducted their examinations on the passage to Liverpool.^57^ When this was not possible, aliens staying in Britain were examined on the landing stage at the dock, allowing officials to concentrate on processing transmigrants on board ship. Overall, the Ministry inspector felt, this led to confusion bordering on pandemonium:

men, women and children, all anxious to get on shore, crowd together round the tables where the Immigration Officers are attempting to carry on their work under conditions of great difficulty … answers to questions put by the Immigration Officer are overheard by a large number of persons and this form of public examination concerning the antecedents of persons entering the country is liable to give rise to resentment on the part of those subjected to such treatment. The shipping companies do not set aside a suitable cabin where the Medical Inspector can conduct the medical examination of aliens. On a case being referred to him he has to go in search of a cabin, and, in the case of a woman, has to persuade a stewardess to be present at the examination^58^

The inspector went on to observe if there was no porthole in the cabin then ‘the examination has to be carried out by artificial light’, which led to some conditions being missed. All this was exacerbated by the sheer noise ‘on board ship incidental to the disembarkation of passengers and the working of machinery [which] in many cases renders examination of the lungs or heart by auditory methods impossible’.^59^ Overall the report creates a picture of the physical chaos of the process of inspection, driven by a series of contingent factors rather than governed by a professionalised medical process.

Dr Hope was aware of the inadequacies of the arrangements and developed alternative plans which included building examination rooms on the landing stage.^60^ This was vetoed by the Ministry inspector who argued that ‘[i]f the Immigration Officers are going to continue to examine aliens on board ship, then a medical examination room ashore will not be of much advantage’. This organisational issue compounded that of expense, with the estimate for the cost of joint medical/customs accommodation standing at £66,000. While the Ministry was not solely against the idea, it required the Treasury to commit to contributing around £3,000 a year which, given the ‘necessity for economy … was out of the question’.^61^

Hope’s suggested alternative, that accommodation be routinely made available on board ship for medical inspections, was dependent on shipping companies agreeing to provide a cabin ‘well lighted with daylight and … situated as to be as far away as possible from the noise of machinery etc., as possible’.^62^ But yet again, finance was an issue. Shipping companies had been hit by the introduction of the US immigration quota system, which had significantly restricted the transatlantic trade, so that by the end of 1920 ‘a number of their vessels [were] laid up, and the Companies [were] not prepared, in the circumstances, to embark upon any scheme involving considerable expenditure’.^63^ The stymieing of both the Ministry’s and Hope’s suggestions demonstrated how neither the Port Sanitary Authority nor the Ministry of Health was able to act independently. Rather, they were required to work within the wider structures and limitations imposed by the immigration service and shipping companies, which in turn were operating within a broader context of economic stringency and American legislation.

The only factor directly under the MOH’s control was his ability to organise his medical staff. Owing to the frequent but irregular arrival of ships, two half-time Assistant MOHs were needed. In order to avoid calling upon the same medical officers for seven days a week, Hope drew on temporary cover provided by the wider pool of medical officers within Liverpool’s public health department.^64^ On top of this extra cover Hope was able to employ a council nurse so he did ‘not have to rely on the somewhat precarious services of a stewardess’.^65^

Overall the picture painted in Liverpool is not of a dynamic state or a modern bureaucracy leaping into action. Instead we gain a sense of a diligent MOH trying to implement a new measure with little institutional or financial support. Subsequent annual reports confirm this impression, showing how little progress had been made, with, for example no examination rooms having been constructed or made available. And behind the ever-present explanation of the need for financial prudence, was the Home Office which was ‘unwilling to exercise any great pressure at the present time … and [did] not wish to arouse opposition’ to its alien immigration powers.^66^

In part then, the lack of progress can be ascribed to the general climate of austerity which hit the country after 1921, with the cuts in government spending affecting central departments as well as local authorities. But beyond this, the failure to develop the necessary resources for medical inspection was due directly to the Home Office’s unwillingness to seek permanent legislation. What was presented as bureaucratic prudence was in fact the Aliens Department’s refusal to jeopardise its powers, and when combined with extreme economy, such ongoing uncertainty prohibited any development of a clear rationale for investment in permanent facilities. Without the backing of the Home Office and the certainty that permanent legislation would justify the expense, the Ministry of Health was unable to challenge the parsimony of the Treasury and insist on investment in new facilities.

On the ground, this meant that medical inspections continued to be carried out on board ship with little change: where improvements had been made it was in the staffing and organisation which Hope had under his control.^67^ Inevitably this had consequences for his staff’s ability to operate a full and effective regime. While all the larger vessels were visited by at least one of the medical inspectors, it remained ‘impossible with only two Medical Officers, having other duties to perform, to visit all the smaller vessels’. This was particularly the case when immigration was dealing with two or three vessels in different parts of the port at the same time. As a result the immigration and medical inspectors developed an informal system between them in which the former performed the first stage of medical inspection and selected aliens who appeared to need further examination. Their selection was guided ‘chiefly by the proposed length of stay, the appearance of the alien and any information disclosed in his papers’.^68^ Although such close cooperation between the two services had been proposed by civil servants, the use of discretion in this way was the opposite of what had been envisaged: here it was immigration inspectors who were making the initial judgement—a judgement based in no small part on ‘appearance’—rather than medical professionals. While we may suspect that medical inspectors would also have used ‘appearance’ as part of a discretionary approach—and hence be subject to biases—it is clear that civil servants had at least expected discretion to be based on medical grounds rather than appearance alone. Here was the reintroduction of the focus of inspection on steerage passengers by another route, one demanded by exigency rather than legislation.

Even a generous assessment of the public health measures being carried out under the Aliens Order, suggests that day-to-day practice failed to match up to either its original wording or the rather more restricted vision developed by Ministry of Health civil servants. Not only was it the case that ‘aliens passed by the Immigration Officer may not necessarily be seen by the Medical Officer’, but it was common practice for vessels coming from Ireland to go straight into dock without ever being seen by any port medical officer. As a result, as one Ministry inspector pointed out it ‘any undesirable alien … who may want to land in this country has only to take passage to, and then come over from, Ireland to one of our ports’.^69^ This was not the diffuse power of the state, but rather its considerable dilution.

Beyond these limitations however, from a public health, if not an immigration, perspective, there was a fundamental flaw in the Aliens Order exposed in the everyday experience of Liverpool. A key presumption behind the Aliens Order was that it was aliens, rather than British or imperial subjects, who were the cause of public health concern. However as Dr Stock, the senior Ministry of Health inspector admitted, trachoma was ‘found amongst British Jews and the poorer Irish, Welsh and Scotch emigrant’ rather than aliens, which created a medically unsatisfactory position. For as he observed, ‘we do not medically inspect the Irish, but in view of the statement that 65% of the Irish emigrants are verminous, I think it would be a good thing if we did’.^70^ The issue was, of course, not a medical one, but a legal one, as medical inspectors could not examine Irish migrants as ‘“alien immigrants”’ as they were ‘still British subjects’. It was possible to deal with ‘lousy Irish emigrants’ under port sanitary regulations, but this would have entailed further pressure on an already overstretched service, as well as opening up the far broader question of the regulation of entry of all imperial subjects.^71^ The strong correlation between certain infectious diseases and Irish migrants made a mockery of the presumption behind the Aliens Act that it was aliens that presented a medical threat to Britain.

Overall, procedures only worked as smoothly as they did in Liverpool because of two factors which were entirely outside the working of the public health system. The first was that in the opinion of the MOHs, compared to pre-war migrants, aliens passing through were generally healthy. So, for example, in the year from 1 April 1921, only 8,516 of the 26,595 aliens landing in Liverpool were medically inspected. Of these, six temporary visitors and seven permanent aliens were issued with medical certificates.^72^ These figures speak both of the limited numbers of aliens inspected—only about one third—and of their relative good health. The reason given for this was that, contrary to the popular image of transmigrants as poverty-stricken Jews, many were in fact returnees from North America having ‘made money’ and returning to visit their home country or re-settle there.^73^

More significant was the fact that transmigrants going to the United States underwent a formalised process of inspection, disinfestation and treatment which was designed to ensure they were free from notifiable diseases prior to embarkation.^74^ This was a largely parallel process carried out jointly by the shipping companies and employees of the US government and driven by American immigration law. By the 1920s it had been in existence for three decades and had developed into an efficient process. By comparison, the newly established British state’s solution to the ‘alien problem’ appeared underfunded, flawed, inefficient, hampered by a recalcitrant Home Office and the legal distinction between aliens and imperial subjects which obstructed any methodical inspection of Irish migrants.

## London

London was Britain’s largest and busiest port, handling vessels from Europe, the empire and the wider world. Of all the country’s ports it had the most developed physical and bureaucratic infrastructure as well as an extensive and long-established body of customs officials; since 1873 it had had a full-time Port MOH and sanitary inspector as well as a hospital ship for quarantine purposes staffed by its own medical officer. Yet, despite the relatively advanced nature of its port regime, those trying to regulate entry into London docks were hampered by the sheer size of the port. Ships docked 25 miles downriver at Gravesend and Tilbury as well as anywhere along the 11 miles of docks and wharves of the port of London itself. Adding to these logistical issues were the significant river tides which dominated shipping movements. So although the river had been divided in two authorities—Gravesend Control and London Docks Control—the ability to board vessels for inspection still largely ‘depend[ed] on the state of the tide’. So while a ship might have been technically under Gravesend Control, ‘[f]requently vessels can only be dealt with [by Customs and Medical Inspectors] when they have passed up the river to the area under the London Docks Control’ if they were taking the tide. Adding to this was the additional difficulty that aliens could ‘arrive on almost any vessel and be landed at almost any place in the river’.^75^

Only one landing station—Gravesend pier—had any infrastructure in place suitable for processing aliens. Here they went from the ship onto a specially provided train, but as in Liverpool, the combination of inadequate facilities and time pressures meant the process was ‘only moderately satisfactory’, not least because ‘there was not much privacy’. When ‘pressed for time’—typically owing to a late boat arrival and imminent train departure—inspections were conducted ‘on the pier instead of waiting until the aliens had cleared their baggage from the Customs and arrived at the Immigration Office’. Matters were made more difficult for the medical officer who, owing to the lack of a telephone or radio, had to be on duty from the time the boat was expected which was ‘frequently … quite a different thing from the time the boat arrives’.^76^

For aliens not landing at Gravesend, medical inspections were carried out aboard while vessels were sailing up the Gravesend Reach. In these cases ships carrying aliens were meant to be boarded by the immigration officer, an assistant medical officer and a customs officer. Normal practice was for the medical inspector to occupy ‘a position near to the Immigration Officer’ where he subjected ‘each alien to close scrutiny while the alien’s papers [we]re under examination by the Immigration Officer’.^77^ While this scheme had the merit of potential efficiency—as the aliens in theory might all be examined and processed before they docked—in practice there were considerable difficulties. There was no customs official on duty at night in the Gravesend Control, so that although aliens could be medically inspected, they were not legally required to tell a medical officer how long they were staying in Britain, nor were the necessary immigration staff present to act on any certificate which might be issued following an examination.^78^

More than this, however, were the practical problems caused by the medical inspector needing to share a launch with immigration officers, whose work was generally completed much faster. The medical inspector was consequently ‘placed in a very awkward predicament’. If he remained on board to complete his examination he could not move on with the other officials to other incoming boats, ‘one or more of which may be from infected ports’, and so ran ‘the risk of allowing a vessel with dangerous infectious disease on board to proceed to London without examination. On the other hand, if he decide[d] to board the other vessels, he [could] not carry out the medical examination of aliens’. Conducting inspections on the water carried with it other logistical issues, including the transportation of a female nurse, whose presence was necessary in order to carry out the inspection of female aliens. However, it was generally agreed, she could not be expected ‘to climb from the launch on to the deck of the vessel by means of a rope ladder’, as this practice was ‘dangerous enough for men, but … quite out of the question for women’. As in Liverpool, the Medical Inspector was often reliant on the active cooperation of a stewardess from the shipping company on board in order to examine the women on board. No surprise then that the first Ministry report on London port’s inspection facilities ‘frankly admitted that it is quite possible for a certain number of aliens to get through without ever having been seen by a Medical Officer’.^79^

In London, as in Liverpool, day-to-day practice revealed a significant gulf between the expectations of the central state and the ability of its agents, at the micro-level, to enact its measures. The MOH of London Port, Dr Willoughby, was an active and conscientious official who wanted to create an effective inspection regime. Supported by Ministry officials, he proposed to increase the number of medical inspectors (to include at least one Yiddish speaker), and to concentrate all alien landings at Tilbury where suitable examination rooms could be constructed. In order for this to happen the Port Medical Officer needed to be granted exceptional powers to require all vessels carrying aliens, or coming from an infected port, to land at Tilbury:^80^

Decency and order could be realised in a way that is impossible on board ship, transport for the Officers is at hand in the ferry and train, a convenient and medically satisfactory examination could be arranged both as regards place and necessary apparatus, the Immigration Officer and the Medical Inspector could be in constant touch during the examination and stopping to land the aliens would perform a wholesome check on ships which bring these [aliens] in [in] ones and twos.^81^

And yet this apparently simply solution, as well as more general infrastructure developments, were not put in place. As late as 1930, the Port Medical Officer was still complaining that ‘it is difficult for the Medical Officers and the Immigration Officers to work in close co-operation, because alien passengers are landed at so many widely separated places in the River and Docks’. There had been some slight improvements however, as regular services from mainland Europe were now habitually boarded by the duty medical officer at Gravesend, Tilbury had seen the construction of some immigration and medical inspection facilities and a telephone had been installed easing communication between different staff members. However, the long-awaited boarding launch for Medical Inspectors had yet to materialise, and the inspection rooms still had no water or lavatory. And the Port MOH made it clear in his 1930 Annual Report that until his department had access to their own vessel there was no possibility of reaching all boats carrying aliens.^82^ Consequently, as in Liverpool, it was often still down to immigration officers to make initial medical assessments, and to ring for assistance in cases where they thought a medical opinion was required.^83^

What were the reasons behind the arguably surprisingly limited development of London’s inspection infrastructure, given the port’s place in global shipping and proximity to the bureaucratic heart of the nation and empire? Once again the role of Haldane Porter, and the Home Office, appears to have been central to retarding development. In what was almost textbook bureaucratic obstructionism, Haldane Porter produced a detailed and obstructionist rationale explaining why any proposals to land aliens at Tilbury or Gravesend had ‘always broken down’: ‘1) expense; 2) there was no place that could be used as a receiving house; and 3) general administration’. He also stressed the role of the shipping companies in the matter, arguing that there was ‘no place where the examination of aliens could be carried on and he was quite certain that the shipping companies would not be prepared to provide premises for the purpose’.^84^ Onto this he heaped further problems:

there is no available sleeping accommodation where aliens could be detained. The most important difficulty, however, was that the vessels would land their passengers at Tilbury and then proceed up the river to London. If, as a result of examination, it was necessary to refuse permission to the aliens to land and to send them back to their destination, it was difficult to provide means by which they could be put on board the vessel once more for the purpose.^85^

The Home Office’s solution to the issue was to provide one overnight immigration officer, and a new motor launch for Immigration Officers which Medical Inspectors could utilise to accompany the Immigration Officers, which would ensure that their ‘work could be carried on in a fairly satisfactory manner’. Assessing these claims it is hard not to conclude that, as in Liverpool, it was the Home Office itself which did not want to take action. Indeed, a Ministry of Health minute from two years later noted gloomily that Haldane Porter continued to insist that, for reasons which were entirely unclear, it was ‘absolutely impossible to carry out the examination of aliens at Tilbury’.^86^

Haldane Porter’s attitude clearly signalled both an unwillingness to invest in any necessary infrastructure, and a blindness to the unsatisfactory nature of his proposed solutions, as the Ministry inspection reports had repeatedly made it clear that no small part of the problem stemmed from sharing transport with the immigration officers. Moreover, suggesting that shipping companies were also resistant to landing at Tilbury was only partly true—while boats which only carried one or two aliens objected on the grounds of inconvenience, when ‘a vessel brings in a considerable number of aliens the Shipping Agents are apparently anxious to make any arrangements necessary for facilitating their rapid disembarkation and transit to destination’.^87^ And indeed, the expanded landing infrastructure at Tilbury dock in the mid-1920s was entirely a response to the demands of the shipping companies, particularly after a return night service from Tilbury to the Continent was introduced in 1926.^88^ Evidence from the Port MOH and the Ministry inspectors strongly suggests that they were both in favour of fully extending facilities at either Gravesend or Tilbury in order that they might properly carry out their work. We are left then with the conclusion that, even setting aside the important issue of economy, the Home Office, for the sake of keeping the Aliens Order actively on the statute books, was unwilling to support investment in the resources necessary to implement its medical terms.

## Conclusion

Judged on its own terms, the medical parts of the Order were a failure. Quietly, in December 1930 new instructions were issued from the Ministry of Health, downgrading the medical section of the Order on the grounds that this was ‘impracticable without causing delay and inconvenience to passenger traffic out of all proportion to the advantages accruing’.^89^ It is perhaps fortunate that Britain did not rely on its functioning to keep Britain free of certain diseases—for not only did the general health of aliens improve over the early part of the twentieth century, after the 1918–19 influenza pandemic, the country did not face any major imported health threats. As with other pieces of legislation generated more through popular sentiment than evidence, the 1920 Aliens Order’s public health measures were undoubtedly ill-conceived. Insisting on focusing attention on aliens rather than all those entering British ports meant ignoring how many of the notifiable conditions were endemic in the British and imperial populations. Indeed, the inconsistencies it raised did not go unnoticed by the medical officers working Liverpool’s docks and vessels. It is possible that the primary value of the inspections was symbolic, acting as an, albeit highly constrained, performance of governmentality at the border rather than actually preventing the entry of disease.

However, in common with the findings of Levene *et al.* and others working on inter-war public health, this article shows that inefficiencies and holes in the system should not be translated into a story of simple failure, as these years did see developments at all of the ports and in the efficiency of inspection procedures. Yet the retarded expansion of facilities in Liverpool and London were quite clearly a result of Home Office intervention, so demonstrating the importance of paying attention to inter-departmental relationships in understanding how policies might be supported or obstructed by the centre. The newly established and notably less powerful Ministry of Health was unable to challenge the dominance of the Home Office personified by Haldane Porter. Such dynamics mattered, as they translated into withholding financial and political support for investment in infrastructure and personnel. And this article has demonstrated how such obstructionism became visible on the ground in a slew of apparently trivial and mundane ways, including the need to share launches, and to conduct examinations under poor lighting or cramped cabins and inadequate offices, and to struggle with the issues posed by a lack of female staff. These then combined with the physical constraints imposed by working to the tides and patterns of shipping to profoundly undermine the state’s ability to enact its own legislation. As a result, those first face-to-face encounters between aliens and the British state might be cursory, chaotic or even completely evaded. This then is revealing of the potential weaknesses of diffuse and complex systems of state power, for without the necessary resources being devolved, power remains at the centre where it exists in theory rather than being enacted and strengthened through day-to-day practice.

What is striking about the extension of port medical procedures is how much they were driven from outside rather than within the state. The transmigrant boarding houses of Liverpool and the development of Tilbury pier and station, were all either stimulated entirely or primarily by the shipping companies and private interests, which were in turn prompted by American, not British, legislation.^90^ While local public health officials worked closely with these bodies, it is clear that they were reacting to, rather than driving, change. Allied to this, the other key development of the period was the growing importance of international standardisation of controls and procedures across borders: Liverpool and London were part of the transatlantic migrant network which stretched from continental ports such as Antwerp to the USA. The presence of State Department doctors in Liverpool’s transmigrant hostels required port medical officers to work within the boundaries set out by American immigration procedures, and meant that local officials needed to develop international professional protocols. The absence of infestations on transmigrants coming from mainland Europe to London was the result of the efficacy of procedures in Hamburg, Antwerp and other ports of departure.^91^

These changes should not detract from the fact that the British state proved itself unable to create a regularised, efficient and consistent port inspection regime. Limited investment in the necessary infrastructure and personnel had effects beyond meaning that physical examinations continued to take place on board vessels in highly unsatisfactory conditions. Refusal to invest in a separate launch in London, or the reliance in Liverpool on using Immigration Officials to make initial assessments, for example, seemed to signal close cooperation between the two departments, but ultimately compromised the professional standing of the medical officers. Unable to work independently, and often reliant on the opinion of non-medical professionals to assess cases for inspection, given the already low professional standing of public health officials, such practices reinforced rather than challenged their ambiguous status in relation to the wider port and immigration structure. The fact that Port MOHs were not able to approve and enact infrastructural improvements, but rather were reliant on shipping companies and the Home Office only reinforced their marginal standing.

Overall we can see that the medical terms of the 1920 Order were compromised because of the considerable material constraints and physical difficulties involved in enforcing them; but in the context of a modern bureaucratic state—and indeed when we think of London, the heart of that state and Empire—this is not enough of an explanation. Its terms also failed because there was a distinct lack of political will, sometimes local, but primarily central, to invest the resources necessary to surmount these obstacles. Indeed, the limitations surrounding the implementation of the Order are a case study in the coming together of local, contingent and incidental factors with bigger structural issues which dogged a modernising and increasingly ambitious state; a state aiming to construct a complex system of regulation comprising multiple agents and processes.

That it did not succeed in doing so leads us to place the everyday failures to consistently implement the medical terms of the Aliens Order within a wider story of the patchy nature of state power. Foucauldian analyses demand attention is paid to the ‘heterogeneous and dispersed microphysics of power’, and how this is diffused, takes specific forms and is exercised in different institutional sites. Yet this article has shown that the medical state was likely to be cursory, inconsistent or even absent, demonstrating that even within the context of a modern bureaucratic system the state might be unable to live up to its own expectations. In part the legitimacy of a state which positions itself as liberal and democratic rests upon an understanding that it acts impartially. Therefore its ability to enforce legislation consistently is a key part of its claim to treat those at or within its borders equally. In contrast a limited state is both in fact, and is experienced as, arbitrary and therefore may be experienced as individually unfair or vicious. For the 1920s, while much was made of the impartial, professional nature of the medical inspector’s role, it is not hard to read off from the accounts of the port inspection regimes that a prosperous businessman with venereal disease was far more likely to pass through without a glance than third class passenger with the same condition, or even something as minor as head lice. Looking at the enforcement and failures of the 1920 Aliens Order is consequently a way for us to explore the impact on individuals of everyday lacunae in the power of the state. More broadly it points to the legislation as forming part of a longer trajectory across the twentieth century of state failure to the live up to the promises of its immigration legislation.

## Funding

Research for this article was supported by the Wellcome Trust [grant number WT097727MA].

## Footnotes

^1^**Aliens Restriction (Amendment) Act 1919** (9 & 10 Geo 5 c 92). The Act gave the power to make Orders, so the substantive regulations were all contained in the 1920 Aliens Order in Council. This extended the provisions of the 1914 Aliens Restriction Order: landing of aliens was limited to named, restricted ports; all aliens over 16 years were to register with the police; the police were charged with monitoring lodging houses and other places where aliens gathered, and were permitted to shut them down. The Order also enabled deportation of aliens already in Britain if they were arrested for a crime; if they were deemed to be encouraging sedition in the armed forces or promoting industrial arrest; or if the Home Secretary decided it was ‘conducive to the public good’.

^2^Quoted in Thomas Roche, *The Key in the Lock. Immigration Control in England from 1066 to the Present Day* (London: J. Murray, 1969), 92; Hansard HC Debates, 15 April 1919, 114, c.2748. Criticisms of the legislation can be found in Harold Laski, ‘Discretionary Power’, *Politica*, 1933, 3, 274; David Cesarani, ‘Anti‐Alienism in England after the First World War’, *Immigrants & Minorities, 1987*, 6, 5–29; Dallal Stevens, *UK Asylum Law and Policy: Historical and Contemporary Perspectives* (London: Sweet and Maxwell, 2004).

^3^The National Archives, Kew, London (hereafter TNA) HO213/1772, Mr Eagleston’s notes for the basis of ‘B’ Division history of the 1939–45 war, 6.

^4^Stevens, *UK Asylum Law and Policy*, 55, quoting Quentin Hogg, HC Debates vol. 776 (22 Jan. 2969), col. 504; see also Cesarani, ‘Anti‐Alienism’. A positive reading of this can be found in Alison Bashford and Jane McAdam, ‘The Right to Asylum: Britain’s 1905 Aliens Act and the Evolution of Refugee Law’, *Law and History Review*, 2014, 32, 309–50. For an introduction to the longer history of immigration legislation see Dallal Stevens, *UK Asylum Law and Policy: Historical and Contemporary Perspectives* (London: Sweet and Maxwell, 2004). See also Bridget Anderson, *Us and Them? The Dangerous Politics of Immigration Control* (Oxford: Oxford University Press, 2013). On the 1905 Act see Bernard Gainer, *The Alien Invasion—The Origins of the Aliens Act of 1905* (New York: Crance Russak, 1972); John Garrard, *The English and Immigration, 1880–1910* (Oxford: Oxford University Press, 1971); David Glover, *Literature, Immigration, and Diaspora in Fin-de-Siècle England: A Cultural History of the 1905 Aliens Ac*t (Cambridge: Cambridge University Press, 2012); Jill Pellew, ‘The Home Office and the Aliens Act 1905’, *The Historical Journal*, 1989, 32, 369–85; David Feldman, ‘The Importance of Being English’, in David Feldman and Gareth Stedman Jones, eds, *Metropolis London—Histories and Representations since 1800* (London and New York: Routledge, 1989); Helena Wray, ‘The Aliens Act and the Immigration Dilemma’, *Journal of Law and Society*, 2006, 33, 302–23.

^5^Laura Tabili, ‘The Construction of Racial Difference in Twentieth-Century Britain: The Special Restriction (Coloured Alien Seamen) Order, 1925’, *Journal of British Studies*, 1994, 33, 54–98.

^6^Article 1 (2) (c) and (d) empowered an immigration officer to refuse entry to any alien deemed ‘a lunatic, idiot, or mentally deficient’ or if ‘certified by a medical inspector that for medical reasons his admission is undesirable’.

^7^Quarantine had been regulated via the Quarantine Act 1825, but only related to ‘plague or other infectious disease or distemper highly dangerous to the health of His Majesty’s subjects’ and to yellow fever. The Act was finally repealed in 1896. The ‘English system’ developed under the 1872 and 1875 Public Health Acts. Together these established Port Sanitary Authorities under a Port Medical Officer of Health (Port MOH), and created a ‘sanitary zone’ of control. This treated passengers suffering from infectious diseases in isolation hospitals, while healthy passengers and contacts were monitored in their place of destination by local MOHs. This controlled infectious diseases while avoiding the costly delays and inconveniences involved in quarantine. See Krista Maglen, *The English System: Quarantine, Immigration and the Making of a Port Sanitary Zone* (Manchester: Manchester University Press, 2014). More generally Peter Baldwin, *Contagion and the State in Europe, 1830–1930* (Cambridge: Cambridge University Press, 1999); and Anne Hardy, ‘Public Health and the Expert: London Medical Officers of Health, 1856–1900’, in Roy MacLeod, ed., *Government and Expertise—Specialists, Administrators and Professionals, 1860–1919* (Cambridge: Cambridge University Press, 1988), 128–42, 135. For the longevity of the English system’s approach see John Welshman, ‘Compulsion, Localism, and Pragmatism: The Micro-Politics of Tuberculosis Screening in the United Kingdom, 1950–1965’, *Social History of Medicine*, 2006, 16, 295–312.

^8^Maglen, *The English System*, 193.

^9^Alan Kraut, *Silent Travelers: Germs, Genes and the ‘Immigrant Menace’* (Baltimore: Johns Hopkins University Press, 1995), ch. 3; Alison Bashford, *Imperial Hygiene. A Critical History of Colonialism, Nationalism and Public Health* 2nd edn (Basingstoke: Palgrave Macmillan, 2014); Howard Markel, *Quarantine! East European Jewish Immigrants and the New York City Epidemics of 1892* (Baltimore: JHU Press, 1999); Lara Marks and Michael Worboys, eds, *Migrants, Minorities and Health: Historical and Contemporary Studies* (London: Routledge, 1997).

^10^Christine Bellamy, *Administering Central–Local Relations, 1871–1919: The Local Government Board in its Fiscal and Cultural Context* (Manchester: Manchester University Press, 1988); Anne Hardy, *The Epidemic Streets: Infectious Disease and the Rise of Preventive Medicine, 1856–1900* (Oxford: Oxford University Press, 1993); Roy McLeod, ‘The Frustrations of State Medicine’, *Medical History*, 1967, 11, 15–40.

^11^See for example Alysa Levene, Martin Powell, John Stewart and Becky Taylor, *Cradle to Grave: Municipal Medicine in Inter-war England and Wales.* (Berne and Oxford: Peter Lang AG, 2011); Jane Lewis, *What Price Community Medicine? The Philosophy, Practice and Politics of Public Health since 1919* (Brighton: Wheatsheaf Books, 1986); Charles Webster, ‘Conflict and Consensus: Explaining the British Health Service’, *Twentieth Century British History*, 1990, 1, 115–51; John Welshman, *Municipal Medicine. Public Health in Twentieth-Century Britain* (Berne and Oxford: Peter Lang AG, 2000); Martin Gorsky, ‘Local Leadership in Public Health: The Role of the Medical Officer of Health in Britain, 1872–1974’, *Journal of Epidemiology and Community Health*, 2007, 61, 468–72.

^12^See for example Levene *et al*, *Cradle to Grave*; Becky Taylor, John Stewart and Martin Powell, ‘Central and Local Government and the Provision of Municipal Medicine, 1919–39’, *English Historical Review*, 2007, 122, 397–426; John Welshman, ‘The Medical Officer of Health in England and Wales, 1900–1974: Watchdog or Lapdog?’, *Journal of Public Health*, 1997, 19, 443–50.

^13^See for example Steve Cohen, Beth Humphries and Ed Mynott, eds, *From Immigration Controls to Welfare Controls* (London: Routledge, 2002) and Colin Holmes, *A Tolerant Country? Immigrants, Refugees and Minorities In Britain* (London: Faber, 1991).

^14^Pellew,‘The Home Office and the Aliens Act, 1905’; and David Feldman, ‘Was the Nineteenth Century a Golden Age for Immigrants?’, in Andreas Fahrmeir *et al.*, eds, *Migration Control in the North Atlantic World: The Evolution of State Practices in Europe and the United States from the French Revolution to the Inter-War Period* (New York, Oxford: Berghahn, 2003), 167–77.

^15^David Feldman, ‘L’immigration, immigrés et l’Etat en Grande Bretagne aux xix et xx siècles’, *Le mouvement sociale*, 1999, 43–60, and his ‘History of Immigration and Border Controls’, History and Policy: Home Office Seminar Series, 12 November 2014, <http://www.historyandpolicy.org/img/news/uploads/dfeldman_immigration_border_control.pdf>, accessed 12 September 2015.

^16^For example the Immigration Act and the Chinese Exclusion Act of 1882 were tightened by the Federal Act of 3 March 1891 (26 Stat.084). This required pre-embarkation and disembarkation health checks, placing the onus on shipping companies to comply. See Kraut, *Silent Travelers*, ch. 3.

^17^Baldwin, *Contagion and the State*; Krista Maglen, ‘The First Line of Defence’, *Social History of Medicine*, 2002, 15, 413–28; and Paul Weindling, ‘American Foundations and the Internationalizing of Public Health’, in Susan Gross Solomon, Lion Murard and Patrick Zylberman, eds, *Shifting Boundaries of Public Health: Europe in the Twentieth Century* (Rochester: Rochester University Press, 2008), 63–86, 64.

^18^Susan Gross Solomon, Lion Murard and Patrick Zylberman, ‘Introduction', in Susan Gross Solomon et al., eds, *Shifting Boundaries of Public Health* (Rochester: Rochester University Press, 2008), 1–20, 9. The Sanitary Conferences worked towards standardizing international quarantine regulations. See M. Marrus, *The Unwanted: European Refugees from the First World War Through the Cold War* (Philadelphia: Temple, 2002), ch. 2; Paul Weindling, *International Health Organisations and Movements, 1918–1939* (Cambridge: Cambridge University Press, 1995); and Anne Sealey, ‘Globalizing the 1926 International Sanitary Convention’, *Journal of Global History*, 2011, 6, 431–55.

^19^Bashford, *Imperial Hygiene*; Marks and Worboys, *Migrants, Minorities and Health*.

^20^Kraut, *Silent Travelers*; Markel, *Quarantine!*. For a contemporary account see William Evans-Gordon, *The Alien Immigrant* (London, 1903).

^21^Maglen, *The English System*, 157. For alternate perspectives see Bernard Harris, ‘Anti‐Alienism, Health and Social Reform in Late Victorian and Edwardian Britain’, *Patterns of Prejudice*, 1997, 31, 3–34; Edward Hope, *Health at the Gateway; Problems and International Obligations of a Seaport City* (Cambridge: The University Press, 1931). For contemporary comments linking aliens and disease see *Report of the Royal Commission on Alien Immigration* (London: HMSO, 1903), evidence of Dr F. A. Tyrell, Mr Lyttelton and Dr Murphy.

^22^The 1905 Act defined an ‘undesirable’ immigrant as ‘a lunatic or an idiot, or owing to any disease or infirmity appears likely to become a charge upon the rate or otherwise a detriment to the public’. Aliens Act, 1905, 5 Edw.7, S.1(3)(b).

^23^Maglen, *The English System*, ch. 5.

^24^TNA, HO213/1772 Mr Eagleston’s notes, 5–6.

^25^David Cesarani, ‘ Anti‐Alienism in England after the First World War’, *Immigrants and Minorities*, 1987, 6, 5–29, 6. See also TNA, CAB/23/7, Minutes of War Cabinet, 10 and 11 July 1918. For a detailed discussion of the passing of the 1919 Act see Daniel Bunting, ‘The 1914 and 1919 Aliens Act’ (unpublished Master’s thesis, Birkbeck, University of London, 2013). On anti-alien sentiment at this time see David French, ‘Spy Fever in Britain, 1900–1915’, *The Historical Journal*, 1978, 21, 355–70; Colin Holmes, *John Bull’s Island*: *Immigration and British Society, 1871–1971* (Basingstoke: Macmillan, 1988); and Paniyos Panayi, *Immigration, Ethnicity and Racism in Britain, 1815–1945* (Manchester: Manchester University Press, 1994).

^26^See Rodney Lowe, *The Official History of the British Civil Service: Reforming the Civil Service, Volume I: The Fulton Years, 1966–81* (London: Routledge, 2011) for an overview of the expansion of the post-1918 state.

^27^Kenneth Morgan, *Consensus and Disunity: The Lloyd George Coalition Government, 1918–22* (Oxford: Oxford University Press, 1986), 82 and 84.

^28^TNA, MH78/89, ‘Morant to Addison’, 10 May 1919.

^29^Steven Stacey, ‘The Ministry of Health, 1919–29: Ideas and Practice in a Government Department’ (unpublished D.Phil. thesis, University of Oxford, 1984), 77. See also Rodney Lowe, *Adjusting to Democracy: The Role of the Ministry of Labour in British Politics, 1916–39* (Oxford: Clarendon, 1986), 9; George Peden, ‘The Treasury as the Central Department of Government, 1919–1939’, *Public Administration*, 1983, 61, 371–85; Charles Webster, ‘Conflict and Consensus: Explaining the British Health Service’, *Twentieth Century British History*, 1990, 1, 142–7.

^30^Taylor *et al.*, ‘Central and Local Government’; Levene *et al.*, *Cradle to Grave*, ch. 4. On similar dynamics within the Ministry of Labour see Rodney Lowe, ‘The Ministry of Labour, 1916–1924: A Graveyard of Social Reform?’, *Public Administration*, 1974, 52, 415–35.

^31^Rodney Lowe, ‘Bureaucracy Triumphant or Denied? The Expansion of the British Civil Service, 1919–1939’, *Public Administration*, 1984, 62, 291–310, 291; and Lowe, *Adjusting to Democracy*.

^32^The Board of Trade, which retained responsibility for health measures relating to alien seamen, is not within the remit of this article, owing to the exclusion of these aliens from the 1920 Order.

^33^Michel Foucault, ‘Naissance de la biopolitique—résumé du cours au Collège de France’, in *Annuaire du Collège de France, 79e année, Histoire des systèmes de pensée, année 1978–1979; Dit et écrits. Vol. III.* (Gallimard, 1979).

^34^Bob Jessop, ‘From Micro-Powers to Governmentality: Foucault’s Work on Statehood, State Formation, Statecraft and State Power’, *Political Geography*, 2006, 26, 34–40.

^35^See for example Thomas Wilson and Hastings Donnan, eds, *Border Identities: Nation and State at International Frontiers* (Cambridge: Cambridge University Press, 1998).

^36^Except for London and Liverpool which both employed a full-time Port MOH by the early twentieth century, the role of Port MOH commonly doubled with that of general MOH and School MOH.

^37^See Levene, *et al.*, *Cradle to Grave*; Welshman, ‘The Medical Officer of Health in England and Wales, 1900–74’, 443–50.

^38^TNA, MH55/873, internal MoH minute, Marchbank to MacLachlan, 10 February 1921.

^39^See Taylor *et al.*, ‘Central and Local Government’.

^40^Ministry of Health officials were Maurice Craig, L. W. Harrison, A. R. MacLachlan, R. J. Reece, W. Willoughby. Haldane Porter, head of the Aliens Department, was the Home Office representative.

^41^TNA, MH55/368, Aliens Order, 1920, Report of Sub-Committee appointed by the Ministry of Health (hereafter MoH), 1st proof, 12 February 1920.

^42^TNA, MH55/368, minute, Robert Morant, 26 February 1920. Emphasis added. The list of conditions varied over time, but generally included: syphilis, gonorrhoea, soft chancre in a communicable stage; pulmonary tuberculosis; leprosy; favus, ringworm, impetigo, scabies; and trachoma. The exclusion of aliens deemed to be lunatic, idiot or mentally deficient was ‘mandatory’. Skin diseases found in conjunction with ‘chronic dirt tolerance’ could lead to exclusion; while some ordinary acute infectious diseases—smallpox, scarlet fever, measles—did not necessarily mean exclusion. Individuals could be given leave to land on the condition they went to an infectious diseases hospital, and presented themselves to the Medical Inspector on their discharge.

^43^TNA, MH55/368, minute, Robert Morant, MoH, 26 February 1920. Emphasis added.

^44^TNA, MH55/368, Aliens Order, 1920, 1st proof, 12 February 1920.

^45^*Ibid*.

^46^*Ibid*. The scheduled ports of entry were London, Dover, Folkestone, Newhaven, Southampton, Plymouth, Bristol, Cardiff, Liverpool, Glasgow, Leith with Granton, Grangemouth, Tyne Ports, Hull, Grimsby, Harwich, Moville and Queenstown; and the airports of Lympne, Felixstowe and Hounslow.

^47^Bunting, ‘The 1914 and 1919 Aliens Acts’. See in particular his commentary on TNA, KV1/67, Ministry of Reconstruction, Report of the Aliens Committee, 25 January 1918. Also TNA, HO367/2, R. M. Morris, ‘Marginalia for Eagleston’, 25 October 1963.

^48^The Vivisection Act 1876 also needed to be annually renewed.

^49^Jill Pellew, *The Home Office, 1848–1914: From Clerks to Bureaucrats* (London: Heinemann Educational, 1982); Roche, *The Key in the Lock*, 92.

^50^TNA, HO213/1772, Mr Eagleston’s notes, 7.

^51^Cesarani, ‘Anti‐Alienism’; TNA, HO367/2 and HO213/1172.

^52^Amy Fairchild, *Science at the Borders: Immigrant Medical Inspection and the Shaping of the Modern Industrial Labor Force* (Baltimore: Johns Hopkins University Press, 2003).

^53^TNA, MH55/840, Report of Hugh MacEwen, 4 November 1920, Liverpool.

^54^Hope was Port MOH to Liverpool from 1894 to 1924 and also established a range of services for seamen, including a dispensary and reciprocal venereal disease treatment scheme with New York. Hope, *Health at the Gateway*, ch. 1. An overview of the seamen’s dispensary is contained in O. P. Arya and J. B. Plumb, ‘Seamen’s Dispensary, Liverpool 1924–1991’, *Genitourinary Medicine, 1992*, 68, 84–9. See also June Clayton, ‘Dr Edward William Hope, Medical Officer of Health for Liverpool, 1894–1924: Development of an International Public Health Authority', <http://www.evolve360.co.uk/Data/10/Docs/13/13Clayton.pdf>, accessed 16 December 2014.

^55^See for example the case of Eastbourne’s MOH, Dr Willoughby in Levene *et al.*, *Cradle to Grave*, ch. 5.

^56^Hope, *Health at the Gateway*, 18.

^57^Queenstown, renamed Cobh in the late 1920s, is in County Cork, Eire.

^58^TNA, MH55/840, Report of Hugh MacEwen, 4 November 1920, Liverpool.

^59^*Ibid*.

^60^TNA, MH55/840, letter from Dr Hope, MOH to MoH, 15 December 1920.

^61^TNA, MH55/840, Report of Hugh MacEwen, 4 November 1920, Liverpool; and minutes of conference between Haldane Porter, MacLachlan and Reece, MoH, 10 December 1920.

^62^TNA, MH55/840, Report of Hugh MacEwen, 4 November 1920, Liverpool.

^63^TNA, MH55/840, Conference between Haldane Porter, MacLachlan and Reece, MoH, 10 December 1920.

^64^TNA, MH55/840, Dr Hope to MoH, 15 December 1920.

^65^TNA, MH55/840, Report of Hugh MacEwen, 4 November 1920, Liverpool.

^66^TNA, MH55/840, Port of Liverpool, interim report, Dr Stock, 28 June 1922.

^67^TNA, MH55/840, interim report, Dr Stock, 28 June 1922 and 9 April 1924.

^68^*Ibid*., 9 April 1924.

^69^TNA, MH55/840, Dr Reece, minute, 19 July 1922.

^70^*Ibid*.

^71^TNA, MH55/840, minute by Marchbank, 21 July 1921. The MOH of Hampstead similarly contrasted the relatively good health status of Austrian and German refugees with the Irish, Scots and Welsh recent migrants to his borough in whom tuberculosis was endemic. See Report of the Medical Officer of Health for Hampstead Borough, 1938, 172–4.

^72^TNA, MH55/840, interim report, Dr Stock, 28 June 1922.

^73^*Ibid*. However return transmigrants were not medically inspected.

^74^*Ibid.* Second class passengers could be inspected on the landing stage.

^75^Gravesend Control extended from Thames Haven to Cross Ness while London Docks Control dealt with the river upstream from Cross Ness as far as London Bridge. Gravesend dealt with continental arrivals and ‘blue water’ vessels; and London Docks with all vessels missed by Gravesend as well as ‘Baltic arrivals from Riga and Danzig, fruit boats from the Canary Islands, Hamburg, Mediterranean arrivals, Soviet vessels from Petrograd or Archangel’. TNA, MH55/852, interim report, Dr Stock, 8 December 1922, and minute by Dr Reece, 11 December 1920.

^76^TNA, MH55/852, interim report, Dr Stock, 8 December 1922.

^77^TNA, MH55/852, Minute, Hugh MacEwen, ‘Medical Examination of Aliens’, 29 July 1920. Aliens staying longer than three months were to be ‘taken into a separate cabin and subjected to a detailed medical examination’.

^78^*Ibid*.

^79^*Ibid*.

^80^TNA, MH55/852, letter from Willoughby, MOH London, to MoH, 30 April 1920, and minute by Dr Reece, MoH, 30 July 1920.

^81^TNA, MH55/852, Memorandum prepared by Dr Willoughby, ‘Examination of Aliens: Port of London’, October 1920.

^82^*Report of the Medical Officer of Health for Port of London*, 1930, 35. Emphasis added.

^83^TNA, MH55/852, minute, 7 February 1927.

^84^*Ibid*, ‘General arrangements for medical inspection of aliens arriving in the Port of London’, 3 December 1920.

^85^*Ibid*.

^86^TNA, MH55/852, minute, Hugh MacEwen, 15 June 1922.

^87^TNA, MH55/852, Port of London, interim report, Dr Stock, 8 December 1922.

^88^TNA, MH55/852, Letter from Stock to Willoughby, 31 December 1926, and reply, 3 January 1927.

^89^*Report of the Medical Officer of Health for Port of London*, 1930, 72.

^90^This was also true of Britain’s largest transmigrant hostel, Atlantic Park, located just outside Southampton. See Kushner and Knox, *Refugees in the Age of Genocide*, ch. 3.

^91^Alison Bashford, *Medicine at the Border: Disease, Globalization and Security, 1850 to the Present* (Basingstoke: Palgrave Macmillan, 2006).

